# Patterns of Ancestry, Signatures of Natural Selection, and Genetic Association with Stature in Western African Pygmies

**DOI:** 10.1371/journal.pgen.1002641

**Published:** 2012-04-26

**Authors:** Joseph P. Jarvis, Laura B. Scheinfeldt, Sameer Soi, Charla Lambert, Larsson Omberg, Bart Ferwerda, Alain Froment, Jean-Marie Bodo, William Beggs, Gabriel Hoffman, Jason Mezey, Sarah A. Tishkoff

**Affiliations:** 1 Department of Genetics, University of Pennsylvania, Philadelphia, Pennsylvania, United States of America; 2 Department of Biological Statistics and Computational Biology, Cornell University, Ithaca, New York, United States of America; 3 UMR 208, IRD-MNHN, Musée de l'Homme, Paris, France; 4 Ministere de la Recherche Scientifique et de l'Innovation, Yaounde, Cameroon; 5 Department of Genetic Medicine, Weill Cornell Medical College, New York, New York, United States of America; 6 Department of Biology, University of Pennsylvania, Philadelphia, Pennsylvania, United States of America; University of Washington, United States of America

## Abstract

African Pygmy groups show a distinctive pattern of phenotypic variation, including short stature, which is thought to reflect past adaptation to a tropical environment. Here, we analyze Illumina 1M SNP array data in three Western Pygmy populations from Cameroon and three neighboring Bantu-speaking agricultural populations with whom they have admixed. We infer genome-wide ancestry, scan for signals of positive selection, and perform targeted genetic association with measured height variation. We identify multiple regions throughout the genome that may have played a role in adaptive evolution, many of which contain loci with roles in growth hormone, insulin, and insulin-like growth factor signaling pathways, as well as immunity and neuroendocrine signaling involved in reproduction and metabolism. The most striking results are found on chromosome 3, which harbors a cluster of selection and association signals between approximately 45 and 60 Mb. This region also includes the positional candidate genes *DOCK3*, which is known to be associated with height variation in Europeans, and *CISH*, a negative regulator of cytokine signaling known to inhibit growth hormone-stimulated *STAT5* signaling. Finally, pathway analysis for genes near the strongest signals of association with height indicates enrichment for loci involved in insulin and insulin-like growth factor signaling.

## Author Summary

Africa is thought to be the location of origin of modern humans within the past 200,000 years and the source of our dispersion across the globe within the past 100,000 years. Africa is also a region of extreme environmental, cultural, linguistic, and phenotypic diversity, and human populations living there show the highest levels of genetic diversity in the world. Yet little is known about the genetic basis of the observed phenotypic variation in Africa or how local adaptation and demography have influenced these patterns in the recent past. Here, we analyze a set of admixing Bantu-speaking agricultural and Western Pygmy hunter-gatherer populations that show extreme differences in stature; Pygmies are ∼17 cm shorter on average than their Bantu neighbors and among the shortest populations globally. Our multifaceted approach identified several genomic regions that may have been targets of natural selection and so may harbor variants underlying the unique anatomy and physiology of Western African Pygmies. One region of chromosome three, in particular, harbors strong signals of natural selection, population differentiation, and association with height. This region also contains a significant association with height in Europeans as well as a candidate gene known to regulate growth hormone signaling.

## Introduction

The term ‘Pygmy’ is applied to human populations whose adult males exhibit an average height of ∼150 cm or less, although thresholds between 140 and 160 cm have been employed [1]. Such groups are found all over the world including Africa, Asia, and the Americas, tend to live in tropical environments, have high levels of pathogen exposure, and practice a predominantly hunting and gathering lifestyle [2]. Studies of African mtDNA diversity suggest a time to most recent common ancestor (TMRCA) between African Pygmies and West African Bantu groups of ∼60–70 thousand years ago (kya) and between Eastern (Efe, Mbuti, Batwa, Babinga, etc.) and Western (Biaka, Baka, Bakola, Bedzan, etc.) Pygmy groups ∼10 to 27 kya [3], [4], [5], [6], [7]. Approximately 4–5 kya, Bantu speaking populations practicing slash-and-burn agriculture expanded into the forest habitats populated by the ancestors of modern Pygmy populations [8]. This secondary contact resulted in admixture, predominantly involving maternal Pygmy and paternal Bantu ancestors that remains on-going [6], as well as a cultural exchange that eliminated the Pygmy's ancestral language [9].

Short stature in human Pygmy populations is thought to be adaptive and its global distribution indicates either ancient common ancestry among tropical hunter-gatherers or extensive convergent evolution [2]. Several selective mechanisms have been proposed to explain a fitness advantage for small body size in tropical, forest-dwelling, hunter-gatherer populations. These include resistance to heat stress under humid forest conditions [2], reduced caloric requirements in a relatively food-limited environment [2], and a life-history trade-off between cessation of growth and early reproduction [1], [10]. However, the timing of the putative selective episodes and emergence of contemporary Pygmy morphology and physiology remain unclear. It is also unclear how the genetic architecture of body size in these groups may differ from that observed in other West African populations [11] and Europeans, in which ∼180 loci explaining ∼10% of the phenotypic variance have been identified [12].

To date, a variety of physiological traits related to stature have been examined in African Pygmy populations [13], [14]. These studies documented normal levels of circulating human growth hormone (*HGH*) but altered glucose homeostasis, insulin secretion, and free fatty acid profiles following *HGH*, glucose, and arginine challenges [13], [14]. These observations suggest peripheral sub-responsiveness to the effects of *HGH* in various tissues. Subsequent work noted a significant difference in overall growth rate in Pygmies only during puberty and suggested a reduced level of *IGF-1*, whose production is stimulated by *HGH*, is more likely responsible for short stature in adults [10], [15], [16]. In addition, both a reduction in *IGF-1* receptor expression and decreased signal transmission in response to physiological concentrations of *IGF-1* were observed in immortalized Pygmy T-cells [16], [17]. The observations of low levels of high-affinity *HGH* binding protein in both African and Philippine Pygmies [18], [19], and a severe under-expression of *HGH* receptor (*HGHR*) in the Bakola Pygmies [20] suggest a deficiency in *HGH* receptor activity contributes to short stature as well.

With the goal of providing a genomic perspective on the adaptive and genetic basis of short stature in Pygmies, we analyzed genetic and phenotypic data in a set of neighboring Western Pygmy (Baka, Bakola, and Bedzan) and Bantu-speaking (Tikar, Ngumba, Lemande) agricultural populations from Cameroon. Our sample includes 67 Pygmy and 58 Bantu individuals genotyped using the Illumina 1M SNP array. Of these, 57 Pygmy and 39 Bantu individuals have corresponding phenotype data for height. We performed several genome-wide scans for positive selection, including the calculation of F_ST_ and locus specific branch length (LSBL) metrics, as well as iHS and XP-EHH analyses. Since regions exhibiting selection signals are likely enriched for functional variants contributing to adaptive differences in phenotype between populations, we summarize the types of loci found in these regions using pathway enrichment analyses. Finally, using both SNP genotypes directly as well as ancestry estimates that facilitate admixture mapping approaches, we performed association tests within candidate regions identified in our selection scans as well as for SNPs near genes in candidate signaling pathways. This multifaceted approach identified several genomic regions showing strong signals of selection and association, some of which contain candidate genes for height variation. Together, our results provide unique insights into both the complex adaptive process that took place in the ancestors of contemporary Western African Pygmies and Bantu agriculturalists and the genetic architecture of short stature in Western African Pygmies.

## Results

### Population Structure

**Figure 1:**
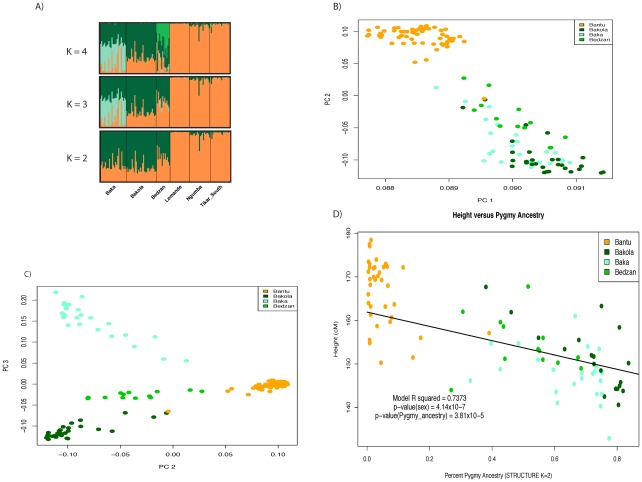
Admixture and population structure. Plots of principal components A) 1 versus 2 and B) 2 versus 3 calculated using 1M SNP data from 67 Pygmy and 58 Bantu samples. The proportion of variance explained by PCs 1, 2, and 3 is 0.245, 0.0192, and 0.0109, respectively. The three Pygmy ethnic groups are clearly distinguished from their neighboring Bantu populations by the first two PCs, while the third differentiates the three Pygmy groups from one another. C) Regression of height on Pygmy ancestry inferred using STRUCTURE (K = 2). Higher levels of Pygmy ancestry are associated with shorter stature. The trend line intersects the intercept from the full model and has a slope equal to the regression coefficient for percent ancestry. D) Visualization of percent ancestry inferred using STRUCTURE at K = 2, 3, and 4.

**Figure 2:**
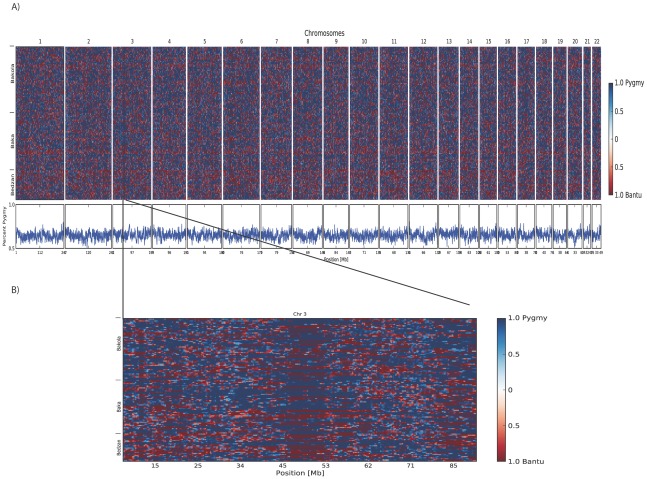
Results from local ancestry inference. A) Genome-wide blocks of Pygmy and Bantu ancestry in Pygmy individuals. B) Blocks of Pygmy and Bantu ancestry in Pygmy individuals for chromosome 3. Shades of blue represent Pygmy ancestry and shades of red admixed Bantu ancestry. Shades are determined by the posterior probability of the estimates; darker colors indicate greater confidence. Each individual is represented by two horizontal rows (one for each haploid genome) and columns represent each window in the genome. Inferred percent Pygmy ancestry for each window is given below. Using SupportMix analysis, we determined the average ancestry block sizes in Pygmies to be 1.7+/−2.4 Mb for Pygmy ancestry, and 3.1+/−4.6 Mb for Bantu ancestry.

We observed extensive and significant genetic and phenotypic differentiation (Figure 1, Figure 2, Figure S1) and varying levels of admixture among the Pygmy and Bantu populations. Average levels of Bantu ancestry, as determined by STRUCTURE (K = 2), in the three Western Pygmy populations were 27% (Bakola), 35% (Baka), and 49% (Bedzan) with individual values ranging from 16–73%. Average levels of Pygmy ancestry in the three Bantu populations were <1% (Lemande), 2% (Tikar), and 7% (Ngumba), with individual values ranging from 0–39%. We also observed a highly significant correlation between ancestry and height (p = 5.047×10^−18^) after correcting for the effect of sex (full model r^2^ = 0.7411, r^2^ for sex = 0.4247; r^2^ for ancestry = 0.3164). In addition, the effect of ancestry remains significant in a model that also includes Pygmy-Bantu ethnicity as a covariate (p = 3.8×10^−5^). These results are consistent with Becker et al. [21] and indicate a strong genetic influence on height. Similar findings were also observed using Pygmy samples only (p_ancestry_ = 0.000216; full model r^2^ = 0.5066; r^2^ sex = 0.3744; r^2^ ancestry = 0.1322) and the independent set of genome-wide microsatellite markers described in Tishkoff et al. [9] (data not shown).

### Inference of Local Ancestry

The relatively high levels of Bantu ancestry in Western African Pygmy individuals suggests admixture mapping approaches will be useful for identifying loci underlying the short stature phenotype [22]. Thus, we inferred ancestry blocks across haploid Pygmy genomes using the machine learning method SupportMix, which does not require an *a priori* admixture model. Consistent with a model of ancient gene flow, we observe very short average tract lengths of Bantu ancestry (3.1+/−4.6 Mb; Figure 2A). No autosomal regions were significantly enriched for either Pygmy or Bantu ancestry (Figure 2A). However, we do observe regions with reduced levels of switching between ancestry blocks. One such region extends across a 7 Mb region from 46 Mb–53 Mb on chromosome 3 (Figure 2B).

### Scans for Selection: F_ST_/LSBL Analyses

We performed several genome-wide scans for selection that have complementary properties for detecting adaptive events on different timescales and under different selective scenarios [23]. We began by calculating pairwise F_ST_ values [24] using the Bantu (N = 44) and Pygmy samples (N = 30) estimated to have the lowest levels of admixture in our STRUCTURE analysis. Next, we identified SNPs that are differentiated specifically on the Pygmy lineage using three-way Locus Specific Branch Length analysis (LSBL [25]) comparing Western Pygmy, Bantu, and Hapmap Maasai (N = 45) populations. These cross-population scans for selection (*e.g.* F_ST_ and LSBL) are expected to be useful for detecting regionally restricted adaptation, including selection on standing variation [26].

**Figure 3:**
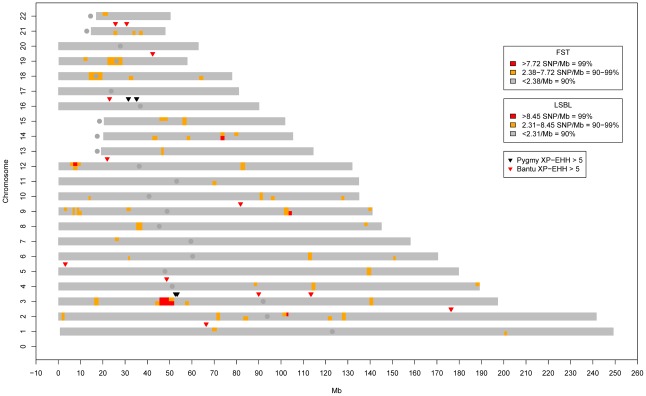
Genome-wide clusters of signals of natural selection in Pygmies. Blocks of high genome-wide density based on SNPs/Mb for F_ST_ (top half of each chromosome) and Pygmy LSBL SNPs (bottom half of each chromosome) are shown. Also shown are XP-EHH signals >5.0 in the Pygmy (Black) and Bantu (Red). The only genomic location showing all three signals occurs on chromosome 3 roughly between 45 and 60 Mb. iHS results for this region are given in Figure 4C and 4D.

**Figure 4:**
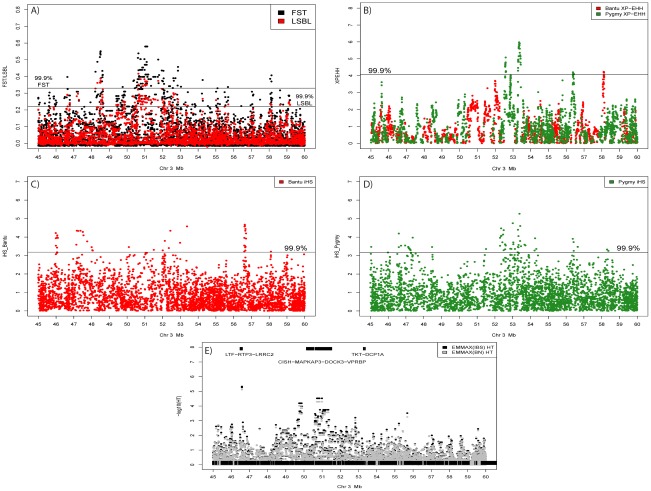
Signals of positive selection and association with height on chromosome 3 between 45 and 60 Mb. A) Results from F_ST_ (Black) and Pygmy LSBL (Red) analysis. The region between ∼50 and 52 Mb contains the largest concentration in the genome of SNPs with scores in the 0.1% tail of the empirical distribution and contains the positional candidate genes *CISH*, *MAPKAP3*, and *DOCK3*. B) Results from XP-EHH analysis. Scores depicted in red show negative values indicating a signal in the Bantu samples, and scores in green show positive values indicating a signal in the Pygmy samples. C) Results from iHS analysis in the Bantu samples. D) Results from iHS analysis in the Pygmy samples. E) Results of association analysis for height (cm) using EMMAX with sex as a covariate. Two suggestive associations are evident. The first, at ∼46.5 Mb, occurs within the coding region of *LRRC2*. The second is a strong enrichment of association signals between ∼49.5 and 51 Mb directly corresponding to Pygmy-specific signals of positive selection in A–D, centered on the three positional candidates *CISH*, *MAPKAP3* and *DOCK3*.

Genome-wide values for pairwise F_ST_ ranged from 0 to 0.58. High F_ST_ SNPs are found on all 22 autosomes, but do not appear randomly distributed across the genome. Rather, of the 947 SNPs in the top 0.1 percentile of the empirical distribution (F_ST_>0.33; Table S1), approximately one third (308) occur in 37 high-density (>2.31/Mb) blocks that average 93 kb in length. Such blocks are defined as a series of four or more high F_ST_ SNPs with less than 100 kb separating each and occur on all chromosomes except 11, 13, 16, 20 and 22. Using these criteria, 33% of the most differentiated loci in the genome are found in a total of just 3.42 Mb. The most striking signal (34.7 high F_ST_ SNPs/Mb) occurs within a 15 Mb region containing 9 separate blocks on chromosome 3 (between 45 and 60 Mb), 7 of which occur between 48 and 53 Mb (Figure 2B, Figure 3, Figure 4A–4E). Interestingly, the highest F_ST_ SNP in the region for which there is genotype data from the CEPH human diversity panel (rs7626978 at position 48505831; F_ST_ = 0.53 [27]) shows a striking global distribution in which the minor allele is most common in the Western and Eastern Pygmy, San, and neighboring populations and is nearly absent outside of Africa (Figure S2).

Genome-wide values for Pygmy-specific LSBL SNPs ranged from 0 to 0.42. High Pygmy LSBL SNPs representing the top 0.1 percentile of the empirical distribution (912 with Pygmy LSBL>0.2215) are found on all 22 autosomes (Table S2) and a large proportion of these (306 SNPs, ∼34%) are found in 38 physical clusters (visualized as regions of density >2.31 high Pygmy LSBL SNPs/Mb in Figure 3) averaging 99.85 kb in length. Many overlap with high F_ST_ clusters, as expected. Nine of the 38 Pygmy-specific LSBL regions also occur on chromosome 3 between 45 and 60 Mb.

In order to test the null hypothesis of a random distribution of signals over the genome, we defined non-overlapping 500 SNP bins (average size ∼1.77 Mb) and assessed the significance of the number of high F_ST_ and high Pygmy LSBL SNPs observed in each using the binomial distribution. We identify 82 and 81 bins with over-representation of high F_ST_ and high Pygmy LSBL SNPs, respectively, that are significant at the p<0.01 level. Among these are three contiguous bins (47560863–49772375 bp, 49773236–52166147 bp, and 52169095–53855083 bp) in the chromosome 3 region showing p-values less than 1×10^−12^ (numbers of high F_ST_ SNPs = 18, 83, and 11; numbers of high LSBL SNPs = 22, 92, 16). Together, these three bins account for 11.8% and 14.3% of all high F_ST_ and high Pygmy LSBL markers in the genome.

F_ST_ and Pygmy-specific LSBL values for X chromosome SNPs are given in Table S3A, S3B). As expected due to its smaller effective population size, these values are higher on average than values calculated for the autosomes. We identify 31 high F_ST_ and 11 Pygmy specific LSBL SNPs within the top 0.1% of X chromosome values, none of which are near obvious candidate genes for height. Because of the distinct demographic history of the X chromosome, we focus further analyses on only autosomal SNPs.

### Scans for Selection: XP-EHH and iHS Analyses

Next, we performed iHS [28] and XP-EHH tests of neutrality [29] in the full set of samples to identify regions that may have been targets of recent selective sweeps within and between populations, respectively. Using an XP-EHH threshold of 4.08 (top 0.1%) we identified 400 SNPs, 54 of which fall in the 45–60 Mb region of chromosome 3. The others are spread over the remaining autosomes with the exceptions of chromosomes 15, 18 and 22. Using an even more stringent threshold of 5.0 (the top 0.02% of the distribution) we identified six Pygmy-specific XP-EHH signals, two of which occur on chromosome 3 between 45 and 60 Mb (Table S4; Figure 3, Figure 4). Using iHS we identified 784 SNPs in the Pygmy population and 807 SNPs in the Bantu population showing signals of selection that are widely distributed across the genome (Tables S5, S6). In the Pygmies, 25 of the top iHS SNPs are again found between 45 and 60 Mb on chromosome 3.

### Pathway Enrichment Analysis of Scans for Positive Selection

In order to summarize the types of loci and explore the potential adaptive genetic architecture implicated by our genome-wide selection scans, we identified all protein coding genes within 100 kb up- and downstream of SNPs showing signatures of selection. Using PANTHER, we then identified pathways that appear enriched in each analysis (Table S7). Significant enrichment for neuro-endocrine signaling and pathways potentially involved in reproduction and thyroid function, including oxytocin receptor mediated signaling and thyrotropin-releasing hormone receptor (TRHR) signaling, was found for genes near high Pygmy LSBL SNPs (Table S7A). Oxytocin plays a role in lactation, parturition, and pair bonding [30] whereas the TRHR signaling pathway plays a role in regulation of the hypothalamic-pituitary–thyroid axis and influences growth, thermo-regulation, reproduction, and immune response [31]. The observation of a relatively low incidence of goiter in Eastern [32] and Western Pygmies (personal communication, B. Hewlett and L. Cordes) relative to neighboring non-Pygmy populations raises the possibility that Pygmies possess a biological adaptation to a low iodine environment [32], [33], consistent with the observation that genes involved in thyroid function are targets of selection in Pygmies [33]. Though there is little significant pathway enrichment for genes near Pygmy-specific XP-EHH and iHS signals (Table S7B–S7C) after correction for multiple tests, several suggestive enrichments are observed for pathways that play a role in immunity and neuroendocrine function. Several of these enrichment categories overlap across different analyses (*e.g.* 5HT2 type (serotonin) receptor mediated signaling, oxytocin receptor mediated signaling, and TRHR signaling pathways). Finally, a more formal test for enrichment of selection signals at SNPs near genes in the candidate *HGH/IGF1* pathway [34] indicated a significant enrichment (p<0.0001) of iHS signals. Similar analyses for F_ST_, LSBL, and XP-EHH were non-significant.

### Signatures of Selection and Association at GWAS Significant SNPs for Height in Non-Africans

A total of 112 of the 211 SNPs significantly associated with height variation in recent GWAS in non-Africans [12] are also present on the 1M chip, passed QC, and are polymorphic in our study populations (Table S8). Interestingly, the highest F_ST_ calculated for a non-African GWAS SNP in our study was 0.23, a value far below our high F_ST_ cutoff, and most (N = 94) showed F_ST_ values<0.10. Only 5 show values greater than the approximate worldwide average of 0.15 and only 7 occur within 100 kb up- and downstream of a high F_ST_ SNP in our study populations. Similarly, the highest Pygmy-specific LSBL value for these markers was 0.14, also far below our 0.1% cutoff. In addition, the maximum absolute value XP-EHH score for a non-African GWAS significant SNP was 2.68, much lower than our cut-off of 4.08. However, one non-African GWAS significant SNP shows an iHS score in the 0.1% most extreme values in the Pygmies (rs16964211 = 3.233). The positional candidate associated with this SNP is *CYP19A1*, a member of the cytochrome P450 superfamily. Overall, these findings are consistent with Pickrell et al [35], which reports little evidence for selection at these loci in Western and Eastern Pygmy populations. Additionally, only four of these GWAS significant SNPs show raw p-values for association with stature less than 0.05 in our combined sample (rs1351394 = 0.01978097, rs724016 = 0.02810565, rs9969804 = 0.04384069, rs4630309 = 0.04465145).

Because of the possibility that the European GWAS associated markers and nearby functional variants identified in non-Africans are unlinked in our African populations, we also compared the positional candidate genes reported in non-African GWAS (rather than the SNPs themselves) to a list of all genes within 100 kb windows up- and downstream of signatures of selection in our study. We observe 69 candidate genes for stature that fall within such windows, including *DOCK3* (33 occur near Pygmy-specific signatures of selection, Table S9). However, 50 candidate genes from GWAS were observed in 2000 randomly sampled, genome-wide 200 kb bins. Thus, observing 69 such loci in the approximately 3500 such bins identified in our analyses does not appear exceptional. These results suggest that the genetic architecture of height in Pygmies differs from that observed in Europeans; however some overlap in specific genes involved remains a likely possibility.

### Admixture Mapping in West African Pygmies

In order to incorporate ancestry information directly into association testing, we performed admixture mapping [22] using EMMAX. Specifically, we tested high confidence ancestry blocks inferred in our Pygmy samples (N = 57) by SupportMix (unpublished data) for association with height variation. This analysis identified several suggestive associations with stature (p<0.002; Table S10), though none show striking FDR-corrected p-values. The strongest (p = 1.40×10^−5^; FDR = 0.304) is observed for a 50 SNP ancestry bin on chromosome 2 approximately 268 kb from the gene *APOB*. Interestingly, studies have demonstrated a correlation between high *APOB* levels and short stature [36].

### Targeted SNP–Based Association Analysis for Height in Western African Pygmies

In an approach adapted from Moore et al. [37], we next performed targeted association tests, first in Pygmy individuals only, for SNPs showing Pygmy-specific signatures of selection (extreme high Pygmy LSBL, Pygmy XP-EHH and Pygmy iHS; N = 2288 SNPs). These marker-based association analyses identified several suggestive associations with height (Table S11A), though they were not significant after multiple tests correction. The marker showing the strongest association (p = 0.0004; FDR = 0.241) is found in a cluster of immunoglobulin lambda loci. The second strongest association (p = 0.0005; FDR = 0.241) occurs near the gene *NDUFA4*. While a direct connection to height variation is not obvious for *NDUFA4*, it is a component of the mitochondrial respiratory chain, which plays a role in metabolic function. The next several strongest associations are found in or near the gene *MAP3K2*, which is involved in NF-kappa B signaling and the regulation of *JNK* and *ERK5* pathways which play a role in growth factor signaling. Additionally, five of the strongest association signals are observed for markers within the 49–53 Mb region of chromosome 3 (Table S11A).

Since growth hormone and *IGF-1* signaling are strongly implicated in Pygmy-specific phenotypic variation by extensive physiological studies in African Pygmies, we next tested SNPs 100 kb up- and downstream of genes in *HGH*, *IGF-1*, and *INS* signaling pathways (N = 40,558) for association with height variation in the Pygmy population. The markers showing the strongest associations with height near *GH-IGF1-INS* genes are in or near the genes *GCK* (p = 6.08×10^−5^; FDR = 0.591), *DUSP4* (p = 7.82×10^−5^; FDR = 0.591), and *IGFALS* (1.20×10^−4^; FDR = 0.591), although none of these associations are significant after correction for multiple tests (FDR<0.05).

### Targeted Association with Height Variation in the Combined Pygmy–Bantu Sample

We next performed association tests in the larger combined Pygmy-Bantu dataset for SNPs showing Pygmy-specific signatures of selection. This both maximizes our sample size and takes advantage of the extensive between-population phenotypic variation. We observed 128 SNPs (Table S11B) showing significant associations after correction for multiple tests (FDR<0.05) and for population substructure. The strongest significant associations are found in or near the genes *NDUFA4* (p = 1.31×10^−5^; FDR = 0.007), ATP2B4 (p = 2.73×10−5; FDR = 0.007), and *DOCK3* (p = 2.99×10^−5^; FDR = 0.007) (Table S11B, Figure 4E). Interestingly, *NDUFA4* is also implicated in the Pygmy-only analysis above. The strongest association signal in *DOCK3* consists of 8 linked SNPs spread over ∼268 kb that are found in the middle of the genomic region on chromosome 3 (45 Mb–60 Mb) displaying multiple signals of positive selection (Figure 4A–4D) and association in the Pygmy population (Table S11A). They also occur ∼85 kb from a marker (rs13088462) showing significant association with height variation in Europeans [12]. Two additional SNPs flanking this European GWAS-associated SNP by ∼4 kb on either side (rs4443210 and rs7638732) also show significant association with height variation in our analysis (p = 0.00018 and 0.00023; FDR = 0.013 and 0.013, respectively).

We next tested SNPs 100 kb up- and downstream of genes in *HGH*, *IGF-1*, and *INS* signaling pathways for association with height variation in our combined Pygmy-Bantu samples (Table S12). The strongest associations are found in or near *CISH* (p = 2.99×10^−5^; FDR = 0.199), *LEPR* (p = 3.36×10^−5^; FDR = 0.199), *EEF2K* (p = 3.44×10^−5^; FDR = 0.199), *PRL* (p = 6.38×10^−5^; FDR = 0.323), and *IFNG* (p = 8.80×10^−5^; FDR = 0.397), though none were significant after FDR correction for multiple tests.

### Pathway Enrichment Analysis in Extreme Genome-Wide Association Signals

Though our small sample size limits power to detect significant associations using full genome-wide association analyses (see Figure S3), we explored potential pathway enrichment for genes 100 kb up- and downstream of markers in the extreme tail of the empirical distribution of genome-wide p-values (the lowest 0.1%) (Table S7D). Consistent with the physiological literature in African Pygmies, the top two pathways that are significantly enriched in the combined Pygmy-Bantu association analysis are the protein kinase B signaling and the mitogen activated protein kinase/MAP kinase cascades of the Insulin/IGF pathway (Table S7D). While PANTHER enrichment for these pathways does not reach statistical significance after Bonferroni correction for multiple tests, the likelihood that these two pathways are the two most enriched by chance is very low, as evaluated using a non-parametric re-sampling test (p = 2×10^−4^).

## Discussion

Our analyses suggest that a complex set of demographic and selective dynamics have shaped genetic and phenotypic variation in West African Pygmies and neighboring agricultural groups. For example, the very short average tract lengths of inferred ancestry we observe (average Bantu tract length of 3.1+/−4.6 Mb) are strikingly different from those seen in simpler admixture scenarios (*e.g.* African Americans). The shorter average Bantu tract length we observe appears to reflect the long history of admixture between Western Pygmy and neighboring Bantu populations that has taken place, possibly since the Bantu expansion into the rain forest several thousand years ago ([8]). Although models assuming a scenario of a single recent pulse of admixture have been developed for inferring the time of admixture based on the distribution of admixture tract lengths [38], more complex models will need to be developed to make inferences about ancient admixture events (e.g. >100 generations) which may fluctuate in rate over time [39].

While the increased genetic resolution offered by short tract lengths may eventually prove useful in fine mapping the genetic basis of complex traits, it also complicates the application of standard approaches to ancestry inference. The novel, model-free SVM approach to ancestry inference we apply here performs well compared to standard approaches in admixed Western Pygmy populations. However further refinement of ancestry inference methods designed to deal with complex admixture histories as well as multiple and/or unknown ancestral populations are needed. Additionally, the development of unbiased African SNP genotyping arrays (*e.g.* those not based on SNPs and patterns of LD identified in non-African populations) are likely to provide novel insights into genome-wide patterns of ancestry and, coupled with increased samples sizes, will facilitate association studies of complex trait variation in African samples.

### Signatures of Selection

Our multiple tests of neutrality, which are sensitive to different selection scenarios and timescales, revealed many signatures of positive selection which are enriched for genes influencing a variety of complex traits with potential fitness implications including body size, thyroid function, immune system function, and reproduction. Although we see evidence for enrichment in *HGH* pathway genes in our Pygmy-specific iHS results, suggesting some selection on these loci after the split between Eastern and Western pygmies (>10 kya), the genome-wide distribution of signals is not consistent with a strong, recent selective sweep at a single locus. Rather, the overall results of our selection scans suggest that the process of local adaptation involved multiple loci that may have been favored at different points in time. Additionally, targets of selection from standing variation or those showing slight changes in allele frequency at multiple loci are challenging to identify. Further development of methods sensitive to these types of complex adaptive signatures will greatly facilitate the analysis of the genetic architecture of adaptation in these and other global populations [40].

### The Chromosome 3:45–60 Mb Region

The region found on chromosome 3 between 45 and 60 Mb shows an overlapping pattern of selection and association signals (Figure 4). The presence of multiple, Pygmy-specific signals of selection using different tests of neutrality could indicate that the region has been repeatedly under selection during the process of local adaptation in Western Pygmy populations. However, the additional presence of Bantu-specific signatures of selection suggests that the region may be more broadly relevant for adaptation in human populations. Indeed, this region has been previously implicated in genome-wide scans for selection in Hapmap populations from Europe, Asia, and Africa [23], [28], [29], [41], [42], [43]. SNPs in this region also show significant association with height variation in both our combined Pygmy-Bantu samples and in recent GWAS studies in Europeans as well as suggestive association in Pygmy samples only (Tables S11 and S12).

Among the most promising positional candidates in the chromosome 3 region are *DOCK3*, a guanine nucleotide exchange factor shown to be associated with height variation in non-African populations [44], *MAPKAP3* a mitogen-activated protein kinase central to several signaling pathways [45], and *CISH*, a negative regulator of cytokine signaling that also inhibits *HGHR* activity [46]. The 709 kb long *DOCK3* gene is expressed specifically in the brain [47] and has no obvious impact on stature, although a SNP within *DOCK3* is associated with height in Europeans [12]. However, ∼63 kb away from *DOCK3*, oriented in the opposite direction, is the 5.4 kb *CISH* gene. *CISH*, a member of the cytokine signaling (*SOCS*) family of proteins, is up-regulated by interleukin-2 (*Il-2*) and plays a critical role in the signaling of cytokines by binding tyrosine residues on activated cytokine receptors, particularly *IL-2R* [48], as well as T-cell differentiation [49]. A recent study demonstrated that genetic variation at *CISH* is associated with susceptibility to bacteremia, malaria, and tuberculosis in several global groups including a Gambian population [48], indicating the important role that *CISH* plays in infectious disease susceptibility. *CISH* also directly inhibits *HGHR* action by blocking the *STAT5* phosphorylation pathway [49], and *CISH* expression is highly regulated by levels of *HGH* expression [50]. Transgenic mice that over-express *CISH* show reduced growth and overall small body size [49].

Sequencing of 5 kb of the *CISH* gene inclusive of the protein coding region, (Figure S4A–S4C) reveals increased diversity in Pygmy individuals relative to the Bantu individuals, but no deviations from neutral expectations. We also did not identify any common population-specific SNPs. However, it is possible that a genetic variant upstream of *CISH*, possibly within or near the *DOCK3* gene, contains a regulatory element that alters *CISH* gene expression in Pygmies.

Although we did not detect any structural variants (SVs) in the region using SNP data (data not shown), *DOCK3* is known to contain inversion polymorphisms in global populations between ∼50.88–50.97 Mb [51]. This observation raises the possibility that an undetected SV could play a role in altering gene expression in the region. Indeed, 8 SNPs among the most strongly associated with stature in our combined Pygmy-Bantu analysis occur in the *DOCK3* gene and are in 100% LD over a ∼268 kb region in both Pygmy and Bantu populations. Specifically, after phasing, genotypes at these SNPs are found as two “scaffold” haplotypes: AAGGGAAG and GGAAACGA, with the “A” form (those with an A at the first SNP, rs6779819) at ∼73% frequency in the Pygmy sample, and ∼36% in the Bantu. These markers also show strong linkage disequilibrium in HapMap European and Asian samples where the same “A” form haplotype is found at 26% and 38% frequency, respectively. Additionally in our Pygmy-Bantu analyses, variation at intervening marker loci define lower-frequency haplotypes, several of which are private (or near private) in the Pygmy population across the region (right half of Figure S5B). This pattern of variation is consistent with the possibility of an ancient inversion encompassing the 8 “scaffold” SNPs that has restricted recombination in the region, with the subsequent accumulation of variation resulting in each sub-type. Moreover, we observe multiple long-range population-specific haplotypes (∼1 Mb in size; Figure S5) in the *CISH/MAPKAP3/DOCK3* region in both the Bantu and Pygmy populations that likely contribute to the high F_ST_ signal (Figure 4A). Additionally, we observed reduced levels of switching between ancestry blocks in the 46 Mb–53 Mb region on chromosome 3 (Figure 2B), consistent with reduced recombination. Future functional studies will be required to determine the magnitude of any *CISH* expression differences between Pygmies and other populations and how these might alter the downstream effects of *HGH* in African Pygmies.

In addition to *CISH*, *MAPKAP3*, and *DOCK3* there are several other positional candidate genes in the chromosome 3 region between 45 and 60 Mb that may have played a role in Western Pygmy adaptation. These include *ERC2*, at position ∼55 Mb, which occurs within 100 kb of signals in all five scans for selection (Table S9). Together with *BSN* located at ∼49 Mb in the chromosome 3 region, *ERC2* appears to help regulate neurotransmitter release [52]. This raises the interesting possibility that an interaction between these loci, possibly related to reproduction, is contributing to the strong Pygmy-specific differentiation in the region. Thus, it is possible that a co-adapted complex of genes in the chromosome 3: 45–60 Mb region has been under selection in the Western Pygmy lineage, as has been observed in other species in regions with reduced recombination due to structural variation [53].

### Pathway Enrichment

The pathway enrichment results from our selection scans and height association results provide suggestive, novel insights into the possible genetic and physiological architecture of adaptation in Western African Pygmies. Signatures of selection in Western Pygmies are often found near genes involved in neuro-endocrine signaling pathways related to reproduction and steroid hormone synthesis, supporting the proposal that differences in the timing of reproduction may partly explain short stature as adults in these populations [1]. Further study of specific genes in these pathways and their expression patterns are likely to yield novel insights about their functional role in adaptation in Pygmies. In addition, the enrichment for genes that play a role in the Insulin/IGF pathway near the strongest signals of genome-wide association further supports a major role for this pathway in the genetic architecture of height in Western African Pygmies.

### Conclusions and Future Studies

Our multi-dimensional approach incorporating ancestry estimation, genome-wide scans for positive selection, and genotype/phenotype association identified several candidate genes and pathways that may contribute to adaptive phenotypes, including short stature, in Western African Pygmy populations. Our results raise the possibility that the adaptive process that produced small body size in Pygmies may be the result of selection for traits other than stature, including early reproduction, metabolism, and immunity, and that the functional variants (possibly regulatory in nature) that are targets of selection may have pleiotropic effects. Indeed, given the extremely high pathogen load and short life span in Pygmies (between 15.6 and 24.3 years [1]) immune function is likely to have been under strong selective pressure in Pygmy populations. Furthermore, many of the loci that play a role in immune response (*e.g.* cytokines) are known to directly influence genes related to *HGH* and insulin/insulin-like growth factor signaling [54]. It is also possible that there have been multiple selective events in the history of the Pygmy populations, at different time periods, that may have contributed to the co-adaptive evolution of loci that play a role in immunity, metabolism, and neuro-endocrine function. Comparison of selection and association signals in Western and Eastern Pygmies will be informative for distinguishing if selection for short stature evolved in an ancestral Pygmy population prior to their divergence within the past ∼25,000 years [3], [4], [5], [6], or whether this trait evolved independently in these populations due to convergent evolution. In addition, future analysis of whole genome sequence, characterization of structural variants, and functional studies of gene expression after challenge with *HGH* and *IGF-1* in appropriate cell types will be particularly informative for identifying genomic variants that play a role in short stature in Pygmy populations.

## Materials and Methods

### Subjects and Samples

Appropriate IRB approval for this project was obtained from both the University of Maryland and the University of Pennsylvania. Prior to sample collection, informed consent was obtained from all research participants, and permits were received from the Ministry of Health and National Committee of Ethics in Cameroon. In total, our study included 132 individuals from six populations residing in Cameroon. Three are Niger-Kordofanian Bantu-speaking hunting and gathering populations: the Baka, Bakola, and Bedzan, and three are neighboring Niger-Kordofanian Bantu-speaking agricultural populations: the Ngumba, Southern Tikar, and Lemande. While the term ‘Pygmy’ has historically been pejorative, more recently it has been used by indigenous groups themselves as well as activist groups working on their behalf [55], [56], [57]. In acknowledgement of this recent trend and in the absence of a better term that encompasses the hunting and gathering peoples from Cameroon, we use ‘Pygmy’ to collectively refer to the set of three populations, the Baka, the Bakola, and the Bedzan, included in our study and their ancestors. Likewise, we will use the term ‘Bantu’ to refer to the three agricultural populations in our study, the Lemande, the Ngumba, and the South Tikar, and their ancestors. Samples were collected in villages in the Eastern Province (Baka and neighboring Bantu groups), Southern and Ocean Provinces (Bakola and neighboring Bantu groups), and Center Provinces (Bedzan and neighboring Bantu population). White cells were isolated in the field from whole blood with a salting out procedure modified from [58] and DNA was extracted in the lab with a Purgene DNA extraction kit (Gentra Systems Inc., Minneapolis, MN). Height measurements as well as self-identified ethnicity, parent and grandparent information were also recorded except in the case of the Lemande for which no phenotypic measurements are available.

### Genotyping and Quality Control

DNA samples were genotyped on the Illumina Human 1M-Duo DNA analysis BeadChip. All DNA samples showed SNP call rates of at least 95%. In order to ensure the quality of genotype calls, SNPs with more than 5% missing data were excluded from analysis. The remaining autosomal dataset includes a total of 1,083,730 SNPs of which 960,497 were polymorphic in our populations of interest. An additional 40,949 X chromosome SNPs exceeded similar quality control metrics and were analyzed separately to determine F_ST_ and Pygmy LSBL values. In order to identify close genetic relatives in the autosomal dataset, we estimated identity by descent (IBD) using PLINK v 1.07 [59]. Seven individuals (4 Bakola, 2 Bedzan, and 1 Tikar South) were closely related to one or more individuals in the sample. After their removal no pairwise estimate of pi-hat exceeded 0.25, a value roughly corresponding to relationships closer than grandparent-grandchild. The remaining set of 125 samples (67 Pygmies and 58 Bantu) served as the basis for subsequent analyses.

### Estimates of Population Structure and Ancestry

In order to evaluate population structure in our sample, we identified a subset of SNPs that maximizes the number of markers while minimizing linkage disequilibrium (LD). Specifically, we removed SNPs within a sliding window (100 SNPs per window and an offset of 50 SNPs) based on the variance inflation factor [59] (VIF = 2). The resulting 30,404 markers were analyzed using STRUCTURE to estimate the proportion of individual ancestry for a range of ancestral clusters, K = 2, 3, and 4 (Figure 1A). Principal components analysis (PCA) was performed using R (Figure 1B, 1C; R Development Core Team [60]).

### Correlation between Global Ancestry and Height Variation

We explored the relationship between global admixture and height variation using linear regression. Estimates of ancestry were analyzed using the following model:
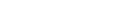
Where G is a co-variate representing sex and P represents the proportion of Pygmy ancestry. Age was initially included but was not significant and so was removed from the final analysis (Figure 1D).

### Pairwise F_ST_ and LSBL analyses

First, using the subset of least-admixed Pygmy (N = 28) and Bantu (N = 40; Baka = 17, Bakola = 13) individuals identified in our STRUCTURE analysis, we calculated pairwise F_ST_ [24] values for all SNPs that passed quality control. Cross-population scans for selection (*e.g.* F_ST_ and LSBL) are expected to be useful for detecting regionally restricted adaptation, including selection on standing variation [26]. SNPs with F_ST_ values greater than 0.3308 represent the 99.9% tail of the empirical distribution and are referred to as “high F_ST_ SNPs”. Next, in order to identify allele frequency differences specific to the Pygmy lineage, we incorporated data from unrelated individuals from the HapMap Phase 3 Maasai sample (N = 45) [61]. We then calculated Pygmy locus-specific branch length (LSBL) values [25] for each SNP that appeared in both datasets. Values greater than 0.2215 represent the 99.9% tail of the empirical distribution and are referred to as “high Pygmy LSBL SNPs”. In order to better visualize both the clustering and concurrence of F_ST_/LSBL signals, we calculated and plotted the density of high F_ST_ and high Pygmy LSBL SNPs per Mb for all non-overlapping 500 SNP windows across the genome (Figure 3). A matrix of average pairwise F_ST_ values between each population is given in Table S13. We were also interested to see if any unusual patterns of population structure exist on the X chromosome, and so analyzed these data separately. The Illumina software package genome studio (Illumina, Inc, San Diego, CA) was used to call SNPs in the female individuals only, and once this was accomplished, males were re-incorporated to establish hemizygous allele calls.

### XP-EHH and iHS Analyses

Using binary executables made publically available by the Pritchard laboratory at the University of Chicago, we performed both cross-population extended haplotype homozygosity (XP-EHH) [29] and integrated haplotype score (iHS) [28] tests of neutrality. The XP-EHH test is most powerful when the selected allele and its associated haplotype are nearly fixed in one population but remain polymorphic or absent in a comparison population. In contrast, iHS is most powerful when a selected haplotype has reached intermediate frequency and is thus useful for detecting partial selective sweeps within a single population [29]. The iHS test has also been shown to be more robust than alternative methods under complex demographic histories and variable recombination rates [62].

SNP genotypes were computationally phased using fastPHASE v 1.4 [63] treating each chromosome independently. Haplotypes that minimized the fastPHASE switch error were used in subsequent analyses, as is recommended for data sets with many markers [63]. A fine-scale recombination map relevant to the African populations included in our study was generated using LDhat version 2.1 [64] and a dataset including a total of 100 unrelated samples. Specifically, we analyzed 25 males and 25 females, from each of two populations in HapMap3 Release 2 [65]: the Yoruba from Ibadan, Nigeria (YRI) and the Luhya from Webuye, Kenya (LWK). HapMap3 Release 2 (January 2009) data were used rather than the more recent HapMap3 Release 3 (May 2010), since these were phased using trios.

LDhat uses a Monte Carlo approach to sample from the posterior distribution of the population genetic parameter 4*N*_e_ *r* for intervals between consecutive markers, where *N*_e_ is the effective population size and *r* is the per-generation recombination rate. The LDhat Markov chain was run for 10,000,000 iterations; 1,000,000 iterations were used as burn-in and only every 5,000^th^ sample was retained to reduce auto-correlation among the posterior samples. This was done for both populations separately. The median of the retained samples was calculated in order to obtain point estimates for the population-specific 4*N*_e_ *r* values. To avoid any population-specific demographic or selection effects in the recombination map, the YRI and LWK estimates were averaged. Using these population-averaged estimates, we computed genetic positions for each SNP in our data in units of 4*N*_e_ *r* that were subsequently used in the XP-EHH and iHS tests. For SNPs present in our data but not in HapMap3, genetic distance was assumed to scale linearly within intervals.

iHS analysis requires ancestral state information. To establish ancestral and derived alleles for all SNPs in our data, we used the human, chimpanzee, orangutan, and rhesus macaque reference alleles in dbSNP131 [66]. The data were downloaded from table snp131OrthoPt2Pa2Rm2 of the University of California at Santa Cruz genome browser [67]. An allele was determined to be ancestral to the human population if the human reference matched 1) the corresponding chimpanzee allele, or 2) the orangutan allele if the chimpanzee allele was missing, or 3) the rhesus macaque allele if both the chimpanzee and orangutan data were missing. Approximately 5% of the SNPs in our data could not be assigned an ancestral state, due mainly to missing data in dbSNP131; this was consistent across all autosomes (data not shown). SNPs with minor allele frequencies less than 5% in either the Pygmy or Bantu populations were removed from the phased dataset in agreement with recent publications [*e.g. 28*]. SNPs with ambiguous ancestral states were also removed from the iHS analysis. A single XP-EHH process comparing the Pygmy sample to the Bantu samples was executed and two independent iHS processes, one for the Pygmy and one for the Bantu samples, were performed. The non-standardized scores returned by the XP-EHH and iHS binary executables were adjusted such that all scores had zero means and unit variances, either with respect to the entire data set (for XP-EHH, as described in reference [29]) or to SNPs with similar derived allele frequencies (for iHS, as described in [28]). The threshold values established from the 99.9% tail of each empirical distribution were as follows: absolute value of XP-EHH = 4.08, iHS_Pygmy = 3.18, and iHS_Bantu = 3.16. This imposes cutoffs that are either comparable or more conservative compared to the values used in previous studies using these tests (*e.g.* [29]; [28]). It is important to note that genomic regions identified using “outlier” approaches are not all expected to be targets of selection. Rather, SNPs within the tails of the empirical distribution are expected to be enriched for linkage with adaptive variants or can be false positives caused by historical demographic events [29]; [28]. We focus primarily on Pygmy-specific signals as these are of greatest interest with respect to identifying candidate loci that play a role in short stature in that population.

### Local Ancestry Inference and Admixture Mapping

Common methods for admixture mapping assume recent admixture, relatively long tracts of LD throughout the genome, and the presence of both reference parental population samples [68]. While methods such as STRUCTURE are able to estimate admixture proportions well globally, they perform poorly with respect to the local ancestry assignments critical to accurate admixture mapping analysis [69]. Other methods such as LAMP [69] do significantly better in this respect, but require knowledge of allele frequencies from both ancestral populations for optimal performance [70]. HAPMIX [71] and PCAdmix [72] have similar requirements. In addition, with the exception of HAPMIX, most methods require unlinked markers. The demographic history of Western African Pygmy populations creates a unique challenge for these methods since admixture is both ancient and on-going. Thus, we expect a wide distribution of haplotype sizes including many that are relatively short. Furthermore, since every Pygmy individual in our sample shows some degree of Bantu admixture, only one of the two ancestral populations is still available and sampled (Bantu). We therefore have applied a novel method of local ancestry inference, SupportMix, that can identify relatively small tract lengths to estimate genome-wide ancestry (unpublished data). Simulations demonstrate this method is more accurate than LAMP for several different degrees of population structure (unpublished data). When LAMP-ANC was performed using information from both ancestral populations and compared to SupportMix using only a single ancestral source population, SupportMix was again more accurate (Figure S6). Thus, SupportMix is an appropriate method to use when ancestral allele frequencies are known for only one ancestral population, as is the case for the Western Pygmies.

Briefly, SupportMix is a two-step process that classifies the ancestral origin of small regions of the genome using a support vector machine (SVM) trained on the ancestral population(s). This is followed by a classification of ancestry with the aid of a Markov model. Specifically, the SVM learns the parameters of the Markov-process and the most probable ancestral state is solved by the forward-backward algorithm over the observed states. As noted above, the complex pattern of admixture observed in our Pygmy samples hinders this training step. To overcome this, an additional SVM was trained at each window on all Pygmy and Bantu samples. Those Pygmy samples most different from the Bantu as measured by the L2 norm from the dividing SVM hyper-plane, were chosen as region-specific “ancestral” Pygmies. This allowed a new support vector machine to be trained to segregate these region-specific “ancestral” individuals from the Bantu. This was then used to classify the remaining Pygmy samples as either ancestrally Bantu or Pygmy for a given genomic region. The proportion of region-specific “ancestral” Pygmies, chosen in each window was the same for every window and was chosen to obtain a global ancestry proportion matching that of STRUCTURE. This agreement is seen in Figure S7A. A window size of 50 consecutive SNPs was sufficient to separate the “ancestral” Pygmies from the Bantu leading to a high posterior probability (69% of windows above 0.9) from the Markov Process. Only these “high confidence” regions of inferred local ancestry were used in subsequent admixture mapping analyses.

### Association Analyses

Association analyses using this dataset are complicated by the inclusion of individuals from two differentiated populations, the complex patterns of admixture within the Pygmies, and our small sample size. These factors greatly reduce the power of a genome-wide association approach (Figure S3). Instead, we used a combination of ancestry-based and marker-based approaches that are expected to produce non-overlapping but complementary information [73] within and between populations. We correct for multiple tests using an analysis-specific false discovery rate (FDR) approach [74], [75] and interpret the most extreme association signals as suggestive. Specifically, FDR values were calculated from the vector of p-values from each association analysis separately. First, we performed admixture mapping using high confidence ancestry blocks inferred by SupportMix in our Pygmy samples (N = 57). Second, in an approach adapted from Moore et al. [37] we performed association tests for two subsets of SNPs for which there are *a priori* expectations of association in Pygmy samples separately. These are 1) SNPs showing Pygmy-specific signatures of selection (extreme high Pygmy LSBL, Pygmy XP-EHH and Pygmy iHS; N = 1823 SNPs) which are expected to be enriched for SNPs in linkage disequilibrium with functional variants that play a role in recent adaptation in Pygmies and 2) SNPs 100 kb up- and downstream of genes in *HGH*, *IGF-1*, and *INS* signaling pathways (N = 40,558) which are strongly implicated in Pygmy-specific phenotypic variation by the extensive physiological studies in African Pygmies described above. Genes with roles in *GH*, *IGF-1* and *INS* signaling were defined via STRING using the criteria: highest confidence, no more than 50 interactors, 200 additional nodes. Third, we performed similar marker-based analyses using all Bantu and Pygmy samples combined in order to maximize both phenotypic and genetic differentiation as well as sample size (N = 125). Finally exploratory genome-wide association analyses were performed using Pygmy samples only, Bantu samples only and the combined dataset (Figure S3).

In all cases, genotype/phenotype association analysis was conducted using EMMAX [76], a mixed-model linear regression approach that corrects for population structure even under cases of extreme differentiation [77], [78]. This method has been successfully used for association mapping in a sample of inbred, and highly sub-structured, dog breeds [77]. Simulations also demonstrate that EMMAX performs extremely well in correcting for population substructure [78]. The additional advantages of this mixed-model approach include simultaneous correction for both relatedness within populations and structure between them via a pair-wise matrix of genetic relationships among individuals. We obtained results using both matrices included in the current distribution of EMMAX (BN and IBS) for both SNP-based and admixture-based analyses. Since they were very similar, we focus on results obtained using the IBS matrix for simplicity. In all analyses, but especially the combined Bantu and Pygmy analysis, EMMAX appears to have provided a reasonable correction for population structure as judged by the resultant Quantile-Quantile plots and the generally low correspondence between high LOD scores and high F_ST_ (Figure S8). Further, by overlaying both *a priori* biological information as well as results from several tests of neutrality our approach, in effect, examines the joint distribution of several test statistics, a scenario under which power is expected to be greatly enhanced.

### Pathway Enrichment Analyses

Several gene lists were generated based on the analyses above and PANTHER was used to summarize and explore types of loci and pathways that are enriched in the tails of the empirical distribution for signals of selection and association. Specifically, genes residing 100 kb up- and downstream of each high F_ST_ and high Pygmy LSBL SNP, as well as those with XP-EHH and iHS scores in the 99.9% of the empirical distribution were identified (100 kb flanking windows were chosen since targets of selection could include regulatory regions). Each was submitted to the PANTHER web tool separately (Table S7A–S7D). We also performed similar enrichment analysis for the lowest 0.1% of the empirical distribution of genome-wide p-values from marker-based association analyses (p<0.001; Table S7A) as well as all p-values<0.05 using ancestry estimates from SupportMix (Table S7B). Pathways passing the significance threshold for analysis-specific Bonferroni corrections for multiple tests are given in bold. In addition, we calculated the probability that the two Insulin/IGF signaling pathways would be the two most enriched in the genes 100 kb up- and downstream of markers in the extreme tail of the empirical distribution of genome-wide p-values (the lowest 0.1%) for association (Table S7D). Specifically, we sampled random sets of genes with HUGO symbols (N = 1248, corresponding to number of genes in the empirical data); only genes with UNIPROT identifiers were retained. UNIPROT identifiers were mapped to all pathways using the Panther database (ftp://ftp.pantherdb.org). We then calculated chi-squared statistics for each pathway in each random list and recorded which showed the two most extreme test statistics. We repeated this procedure 10,000 times and identified either the Insulin/IGF pathway-protein kinase B signaling cascade or the Insulin/IGF pathway-mitogen activated protein kinase kinase/MAP kinase cascade as one of the two most enriched only twice (p = 2×10^−4^).

In order to more directly quantify the potential enrichment of selection and association signals in *HGH* pathway genes, we used an approach described by Eleftherohorinou et al [34]. Specifically we generated a cumulative test statistic, *CA_test_ ∑_s ∈HGH_ β_g_*, where s is the index of all SNPs in the *HGH* pathway and β is the test-statistic (score) estimated for each SNP in a given analysis. If there were an enrichment of signals in the *HGH* pathway, we would expect *CA_test_* to significantly deviate from 0. To assess this, we used a genome-wide re-sampling approach wherein the same number of SNPs found in the *HGH* pathway was randomly sampled with replacement from our set of genome-wide SNPs. We then calculated a new cumulative statistic *CA_rand_*. This procedure was repeated 10,000 times to create a null distribution for each analysis. A skew-normal distribution was fit to the null using maximum likelihood, as described in Eleftherohorinou et al. [34]. A p-value for the observed statistic was calculated using the best-fit null skew-normal distribution.

### CISH Re-Sequencing

We re-sequenced a 5.4 kb region encompassing the CISH gene and its promoter in 19 Bantu (6 Lemande, 7 Tikar South, and 6 Ngumba) and 23 Pygmy (9 Baka, 8 Bakola, and 6 Bedzan). We first amplified the entire region using the two primer sets: forward_1 5′-CCTAGAGGGTCACCTATAACCTACAC-3′, reverse_1 5′-CTTGCTGCTTATCCTCGTCCTTAC-3′ and forward_2 5′-CCTCTGAGAGACACTCCTATCCAT-3′, reverse_2 5′-CCTGCTGTCTATCCTCGTCCTTAC-3′ (Figure S4A, S4B). Individual PCR amplifications were visualized with electrophoresis in a 1% agarose gel and sequenced with the Big Dye Terminator cycle sequence kit (Applied Biosystems, Foster City, CA) and run on an ABI prism 3730XL (Applied Biosystem, Foster City, CA,). For primers used for sequencing see the legend of Figure S4. We performed additional sequencing of a 1.2 kb region to evaluate the reported functional single nucleotide polymorphisms −639, −292 and −163, described by [48] using the primers: forward 5′-GTCCGCATAACGGGAGCAACAC -3′ and reverse 5′-CGCTTACCCCTGAACGCAGAGGACC -3′. CISH sequence results were analyzed and evaluated with the software package Sequencher (http://www.genecodes.com/). Haplotypes were estimated with the software package Phase [79], [80], [81]. Network analysis was performed using the program Network (http://www.fluxus-engineering.com) using the median joining methodology (Figure S4C) [82]. DNAsp (http://www.ub.edu/dnasp/) was used for calculating nucleotide diversity and Tajima's D (Figure S4D) [83].

## Supporting Information

Figure S1 Phenotypic variation for height. Height distributions in A) the combined Pygmy-Bantu sample, B,C) males and females separately and D,E) ethnic groups separately.
(EPS)

Figure S2 Global frequencies of rs7626978. World-wide frequencies of rs7626978, the most structured SNP in the Chromosome 3: 45–60 Mb region that was also genotyped in the Human Genome Diversity Panel (HGDP). Despite the fact that this SNP was likely identified in the Hapmap Yoruba population, it is most common in Pygmy and San hunter-gatherer and neighboring populations.
(EPS)

Figure S3 QQ plots for within-Population Genome-wide Association Using EMMAX. Genome-wide QQ-plots of association results for A) Pygmy samples only and B) Bantu samples only. A genome-wide approach is expected to be underpowered given our limited number of samples. Indeed, no genome-wide significant associations are observed in Pygmy samples only. However, analysis of unrelated Bantu individuals only (N = 39 with height data) yields both a genome-wide significant association (1.31×10^−8^) and a seven-fold enrichment of p-values less than 10^−6^ (expected = 941,183 polymorphic markers×10^−6^ = 0.941; observed = 7, reduced from 10 due to linkage). The genome-wide significant association appears at three tightly linked SNPs (rs13627, rs2189526, rs3815170) located on the short arm of chromosome 19 within the coding region of the gene *GRIN3B*, an ionotropic glutamate receptor. While this region is not identified in the F_ST_, LSBL or XP-EHH results, it does show evidence of an extended haplotype in Pygmies using iHS. The strong statistical signal of association results from the 8 shortest Bantu individuals being heterozygous for the three markers. Interestingly, marker genotypes are at roughly equal frequencies in both Pygmy and Bantu samples. One possible interpretation of this result is that a short-stature haplotype from the Pygmy population is segregating in our Bantu sample. However, the eight individuals show varying haplotypes in the 100 SNP windows surrounding *GRIN3B*. Future re-sequencing of this candidate gene, however, may well reveal a protein coding or regulatory variant with functional consequences. We also observe a suggestive association near *GRIN3B* in the marker-based Pygmy/Bantu association analysis (p = 1.10×10^−5^) and we observe a suggestive association (p = 1.46×10^−4^) near GRIN3A in the ancestry-based analysis. These results raise the possibility that glutamate signaling could play a role in stature in these populations.
(EPS)

Figure S4 Re-sequencing results for *CISH*. Sequencing of the PCR amplification product was performed with the following primers: F_seq_I: GGTTGGTGGGACCCTTTATT; F_seq_II: CTCCACTCACTGCTCATCCA; F_seq_III: GGCAGAAGGCACGTTCTTAG; F_seq_IV: CTGACTGTACGGGGCAATCT; F_seq_V: ATTGTTGAGGGGTAAG; F_seq_VI: CCACTGCAGTTCTGCTAGGTC; F_seq_VII: CCAAATCGCAAGTGGAGAAT; F_seq_VIII: ACCGGACAGTCTGACTTTGG; R_seq_I: ACCAGCCCTGACTTCTCTGA; R_seq_II: CCCCATTGAGGGCTCTGTA; R_seq_III: GCCTTGGTTCCAGCAAGATG; R_seq_IV: CTTGCTCTTGCTGGCTCTTC; R_seq_V: TGACAGCGTGAACAGGTAGC; R_seq_VI: GCCACCTTGATTGTTTCCAT; R_seq_VII: GGTGGACAGTGGCCTCTAAA; R_seq_VIII: GGCTGGGGAAGATGACACTA; F_seq_v: TGCAGGACTCTCTCTCAGCA; F_seq_w: GAGTTCCACCGCGAGATAAG; F_seq_x: CACTGCCTCTCAGTCCCTGT; R_seq_v: CTCTTATCTCGCGGTGGAAC; R_seq_w: TGAACGCAGAGGACCATGT. A) Gene structure of the re-sequenced region. B) SNP genotype calls for each sequenced individual. C) Phased haplotype network for the re-sequenced region. Circles depict haplotypes and their relative sizes represent observed frequencies. Yellow indicates the proportion of each found in the Bantu and blue the proportion found in Pygmies. The black circle in the lower right represents the chimpanzee sequence. Red numbers along edges correspond to segregating sites that differ between nodes. Mv1–mv6 are variants not present in the sample. No significant differences in height were detected for any set of haplotypes. D) Nucleotide diversity and Taijma's D for the combined dataset as well as by population.
(EPS)

Figure S5 Visualization of fastPHASE-inferred haplotypes. Plots exclude SNPs with minor allele frequencies less than 0.1 and are centered on rs107457, the marker showing the highest LSBL value in the chromosome 3 region of interest. A) unsorted visualization B) sorted by haplotype within Pygmy and Bantu groupings. In order to visualize the haplotype structure surrounding SNPs of interest, a custom script was written in R to plot exact haplotype matching for fastPHASE-inferred haplotypes centered on a given SNP. Markers with minor allele frequencies less than 0.1 were excluded. Given a specified SNP of interest and window size (in number of SNPs up- and downstream), the top 10 most frequent haplotypes in the window were identified. These “templates” were assigned the following colors from most to least frequent: black, grey, red, orange, yellow, dark green, light green, dark blue, blue, and turquoise. Next, building outward from the specified SNP, each haplotype in each individual was matched to one of the 10 templates and colored accordingly. Haplotype matches are assessed one SNP at a time first to the left and then to the right of the marker of interest. In the event that an individual haplotype matches multiple templates over the interval at hand, the color representing the most frequent in the window as a whole was retained. Any haplotype stopped being assigned a color at the point that it failed to match one of the templates.
(EPS)

Figure S6 Comparison between SupportMix and LAMPANC using simulated admixture. In-silico admixed HapMap3 individuals between Yoruba (YRI), French (CEU) and Yoruba, Maasai (MKK) were examined with SupportMix using only the Yoruba as an ancestral population. Both ancestral populations were used in LAMPANC. The error bars represent one standard deviation in accuracy across the 25 individuals tested. Specifically, 25 individuals of Yoruba and French ancestry and 25 individuals of Yoruba and Maasai ancestry were generated in-silico using the phased HapMap3 data. The admixture was generated using the same method as described in^32^. SupportMix was carried out exactly as is described the main text and LAMP was run in LAMPANC mode using the allele frequencies of both ancestral populations. It is clear that even when LAMPANC is given more information (allele frequencies for both ancestral populations) it is less accurate in calling ancestry than SupportMix.
(EPS)

Figure S7 Results from SupportMix analysis of local ancestry. A) Average Pygmy ancestry per individual as estimated by SupportMix and STRUCTURE. The STRUCTURE estimates were obtained from a thinned version of the SNP dataset. B) Plot of ancestry tract lengths as obtained by SupportMix with Pygmy tracts averaging 1.7+/−2.4 Mb and Bantu tracks 3.1+/−4.6 Mb. The slight hump at 120 kb represents the average window length and represents a lower bound on the possible tract lengths observable with SupportMix. C) Quantile-Quantile plot of p-values from admixture mapping for Pygmy height obtained from ancestry assignments using SupportMix and EMMAX corrected for population structure using an identity by state (IBS) kernel matrix. D) Genome-wide p-values from admixture mapping for Pygmy height. Each dot represents the p-value as obtained from associating height on ancestry for a window of 50 consecutive SNPs using EMMAX corrected with a kernel matrix representing IBS.
(EPS)

Figure S8 Performance of EMMAX in correcting for population structure. A) QQ plot depicting the severe, upward bias in genome-wide −log10(p) values when no correction for population structure is applied. B) QQ plot of −log10(p) values from a fixed-effect linear regression including overall STRUCTURE estimate of Pygmy ancestry to correct for population structure. C) QQ plot of genome-wide results obtained via EMMAX showing excellent control of p-value inflation due to population structure. D) Hexagonal binning plot of the relationship between p-value and F_ST_ in the genome-wide results from EMMAX. SNPs showing the lowest p-values (surrounded by the black box) are essentially evenly distributed over the full range of F_ST_ values. There also appears to be a qualitatively higher density of low p-values for lower values of F_ST_ further indicating a biologically negligible relationship between F_ST_ and association with height. E) Table showing the low correlation of F_ST_ with p-value both genome-wide and in the subset of high F_ST_/LSBL SNPs. F_ST_ and p-values for SNPs tested for association using EMMAX are only very weakly correlated (Spearman's Rank correlation genome-wide = −0.14; Pearson's correlation coefficient of −0.17, −0.2 (r) and −0.22 (rho) for high F_ST_ and high LSBL SNPs only). Thus, we do not see a strong correlation between SNPs with high differentiation and low p-values in association tests, as expected if population structure alone were driving the global enrichment for low p-values.
(EPS)

Table S1 High F_ST_ SNPs. Position information, F_ST_ and association results for the top 100 markers in the empirical distribution of autosomal F_ST_ values. Raw p-values for association with height using the IBS matrix from EMMAX in the combined Pygmy-Bantu sample and analysis-specific Benjamini-Hochberg FDR corrected p-values are given.
(XLSX)

Table S2 High Pygmy LSBL SNPs. Position information, LSBL and association results for the top 100 SNPs in the empirical distribution of autosomal LSBL values. Raw p-values for association with height using the IBS matrix from EMMAX in the combined Pygmy-Bantu sample and analysis-specific Benjamini-Hochberg FDR corrected p-values are given.
(XLSX)

Table S3 X Chromosome SNPs with high F_ST_ and LSBL values A) Position information and F_ST_ values for SNPs in the 99.9% tail of the X-chromosome specific empirical distribution of F_ST_ and B) position information and Pygmy LSBL values for SNPs in the 99.9% tail of the X-chromosome specific empirical distribution of LSBL values.
(XLSX)

Table S4 Extreme XP-EHH SNPs. Position information, IHH scores for both Pygmy and Bantu groupings, raw and standardized XP-EHH scores and association results for the top 100 SNPs in the empirical distribution of autosomal XP-EHH values. Raw p-values for association with height using the IBS matrix from EMMAX in the combined Pygmy-Bantu sample and analysis-specific Benjamini-Hochberg FDR corrected p-values are given.
(XLSX)

Table S5 Signals of selection from iHS analysis in Pygmy samples. Position information, iHS score, the number of SNPs on which it is based, and association results for the top 100 markers showing scores in the empirical distribution of autosomal iHS scores in Pygmy individuals. Raw p-values for association with height using the IBS matrix from EMMAX in the combined Pygmy-Bantu sample and analysis-specific Benjamini-Hochberg FDR corrected p-values are given.
(XLSX)

Table S6 Signals of selection from iHS analysis in Bantu samples. Position information, iHS score, the number of SNPs on which it is based, and association results for the top 100 markers showing scores in the empirical distribution of autosomal iHS scores in Bantu individuals. Raw p-values for association with height using the IBS matrix from EMMAX in the combined Pygmy-Bantu sample and analysis-specific Benjamini-Hochberg FDR corrected p-values are given.
(XLSX)

Table S7 PANTHER enrichment analysis. Results are given for genes residing 100 kb up- and downstream of signatures of positive selection and association. Significant results for enrichment near SNPs in the 99.9% tails of the empirical distribution for A) high Pygmy LSBL SNPs, B) XP-EHH SNPs in Pygmies, C) iHS SNPs in Pygmies, D) enrichment for genes 100 kb up- and downstream of SNPs showing raw p-values in the genome-wide EMMAX analysis less than 0.001 in the combined Pygmy-Bantu dataset, E) raw p-values less than 0.001 in the Pygmy-only SNP-based association analyses and F) for similar regions surrounding SupportMix bins with associations less than 0.05. The Insulin/IGF pathway-protein kinase B signaling cascade, as well as several other pathways involved in immunity, glutamate receptor signaling, metabolism, neuro-endocrine signaling, and reproduction appear enriched in admixture association analysis within Pygmies. Analysis-specific bonferroni corrections for multiple tests were calculated by dividing 0.05 by the total number of pathways actually tested, less those pathways that include only 1 gene. All pathways showing raw p-values for enrichment less than 0.05 are shown and those meeting the appropriate multiple test correction threshold are given in bold.
(XLSX)

Table S8 Selection, association and GWAS results. Results from scans of selection and association analysis in the combined Pygmy-Bantu sample at the 112 SNPs identified in recent GWAS studies of height in non-Africans that were also included in our study. Values for F_ST_, LSBL, XPEHH, iHS and association with height using the IBS matrix in the combined Pygmy-Bantu sample are given.
(XLSX)

Table S9 Genes showing signatures of selection and/or association with height. Summary of results for all genes 100 kb up- and downstream of SNPs showing both significant signals of positive selection and potential association in one or more of our mapping analyses. Significant results are indicated with an ‘x’. Identification of a candidate gene for height via GWAS in non-Africans and membership in various pathways defined using STRING are also shown.
(XLSX)

Table S10 Admixture mapping results. Association results for the 100 most associated bins using high confidence ancestry estimates from SupportMix. rsID and position information correspond to the first SNP in each 50 SNP bin. Scores and raw p-values using the IBS matrix in EMMAX and analysis-specific FDR corrected p-values along with all genes 100 kb up- and downstream of each of the 50 strongest associations are given.
(XLSX)

Table S11 Top association hits with height for top SNPs in scans for Pygmy-Specific signatures of selection. Position information and association results for SNPs showing both Pygmy-Specific autosomal signatures of selection (LSBL, iHS for Pygmy samples only, and positive XP-EHH) and suggestive association with height in A) the Pygmy samples only (p<0.01) and B) the combined Pygmy-Bantu sample (FDR<0.05). Analysis-specific Benjamini-Hochberg FDR corrected values are also given. Interestingly, in addition to Insulin/IGF signaling, the thyrotropin-releasing hormone receptor and oxytocin receptor mediated signaling pathways appear to be enriched in an analysis of Pygmy samples only.
(XLSX)

Table S12 Top SNPs associated with height near genes involved in the *HGH-IGF1-INS* signaling pathway. The 100 most significantly associated SNPs 100 kb up- and downstream of genes with roles in *GH*, *IGF-1* and *INS* signaling defined via STRING (highest confidence, no more than 50 interactors, 200 additional nodes). Gene name, position information, raw p-values from association analysis for height (IBS matrix) in the combined Pygmy-Bantu sample and in the Pygmy sample only, are given along with analysis-specific FDR values.
(XLSX)

Table S13 Table of average pairwise F_ST_ values for all six populations. SNPs with F_ST_ = 0 were omitted from the calculation.
(XLSX)
